# COVID GEAS: COVID-19 National Survey in Patients With Systemic Autoimmune Diseases

**DOI:** 10.3389/fmed.2021.808608

**Published:** 2022-01-27

**Authors:** Borja Del Carmelo Gracia, Luis Sáez, Lucio Pallarés, Jose Velilla, Adela Marín, Luis Martinez-Lostao, Carmen Pilar Simeón, Patricia Fanlo

**Affiliations:** ^1^Sytemic Autoimmune Diseases Unit, Internal Medicine Department, Lozano Blesa University Clinical Hospital, Zaragoza, Spain; ^2^Sytemic Autoimmune Diseases Unit, Internal Medicine Department, Miguel Servet University Clinical Hospital, Zaragoza, Spain; ^3^Sytemic Autoimmune Diseases Unit, Internal Medicine Department, Son Espases University Clinical Hospital, Palma de Mallorca, Spain; ^4^Immunology Department, Lozano Blesa University Hospital, Zaragoza, Spain; ^5^Systemic Autoimmune Diseases Unit, Internal Medicine Department, Vall d'Hebron University Hospital, Barcelona, Spain; ^6^Systemic Autoimmune Diseases Unit, Internal Medicine Department, Universitary Complex of Navarra, Pamplona, Spain

**Keywords:** COVID-19 infection, SARS-CoV-2, systemic autoimmune disease, survey, systemic erythematosus lupus, corticosteroids, anti-TNF

## Abstract

**Objectives:**

COVID-19 outcomes in population with systemic autoimmune diseases (SAD) remain poorly understood. The aim was to examine demographic and clinical factors associated with COVID-19 infection in people with rheumatic disease.

**Methods:**

Two phases cross-sectional survey of individuals with rheumatic disease in April 2020 and October 2020. COVID infection, severity of disease, age, sex, smoking status, underlying rheumatic disease diagnosis, comorbidities and rheumatic disease medications taken immediately prior to infection were analyzed.

**Results:**

A total of 1,529 individuals with autoimmunity disease diagnosis were included. Out of 50 positive patients, 21 required telephone medical assistance, 16 received assessment by primary care physician, 9 were evaluated in Emergency Department and 4 patient required hospitalization. Multivariate analysis was performed without obtaining differences in any of the systemic autoimmune diseases. Regarding the treatments, significant differences were found (*p* 0.011) in the treatment with anti-TNF-alpha agents with OR 3.422 (1.322–8.858) and a trend to significance (*p* 0.094) was observed in patients receiving mycophenolate treatment [OR 2.016 (0.996–4-081)].

**Conclusions:**

Anti-TNF-alpha treatment was associated with more than 3-fold risk of suffering from SARS-CoV-2 infection, although in all cases infection was mild. Cumulative incidence in patients with SAD was up to 5 times higher than general population but with great differences between autoimmune diseases.

## Key Messages

- Cumulative incidence of SARS-CoV-2 infection in autoimmune population is up to five times higher than general population.
- Anti-TNF-alpha treatment may be associated with a more than 3-fold risk of suffering from SARS-CoV-2 infection.

## Introduction

In 12 March 2020, the World Health Organization (WHO) declared the global outbreak of the severe acute respiratory syndrome coronavirus 2 (SARS-CoV-2) disease 2019 (COVID-19) a pandemic. The main symptoms of respiratory infection include fever and cough in 88.5 and 68.6% of the patients, respectively (1–3). The presence and the number of comorbidities (e.g., arterial hypertension, coronary heart disease), age and lifestyle factors such as smoking appear to have deteriorating effects on the course of the infection (3).

COVID-19 is a serious disease in different groups of patients, and today we know that this severity is due to the hypersensitivity immune response that the virus produces at the pulmonary and systemic level.

Secondary to the presence of comorbidities and mechanism of immune hyperresponsiveness, patients with systemic autoimmunity diseases (SAD) may face a particular risk as their disease. On one side, these patients may be associated with an increased risk of infections due to immunosuppression (4, 5) and on the other, immunosuppression itself can positively or negatively alter the abnormal immune response that seems to be responsible for the most severe disease complications such as interstitial pneumonia (6).

Due to robust knowledge of the course of SARS-CoV-2 infection in patients with SAD is scarce, scientific evidence-based recommendations for the management of COVID-19 in patients with rheumatic disorders and anti-rheumatic treatments are limited (7).

The Spanish group of Autoimmunity diseases (GEAS) developed at an early-stage first concise recommendations for the management of patients with SAD during the COVID- 19 pandemic. Interruption or reduction of immunosuppressive treatment was not recommended as this might result in relapses or flares, that consequently could require the increase of amount of immunosuppressive therapy (e.g., additional glucocorticoids and/or immunosuppressive therapy).

Incidence, course of COVID-19 and including lethal outcomes, vary considerably in different cohorts according to pre-existing conditions and healthcare systems. Investigation of special disease groups may contribute to a better understanding of the role of the immune system regarding the risk to get infected or to develop a more severe course of COVID-19. Based on the clinical information published to date from the outbreak caused by coronaviruses, there is no overwhelming evidence that patients with rheumatic diseases are at an increased risk compared with other kinds of patients (8–10).

Therefore, patients with SAD, who are treated with different types, combinations and dosages of immunomodulatory therapies represent an interesting population to collect data regarding SARS- CoV-2 infection.

Registries with a large number of case reports are required to answer the question of whether antirheumatic drugs increase or decrease the risk for a severe course of SARS-CoV-2 infection. As necessary data cannot be extracted from clinical charts or health insurance records, GEAS decided to establish a web-survey, which allows a rapid and timely collection of patient information of autoimmunity patients in real life in Spain. This web-survey let us to analyze the real incidence and clinical course of SARS-CoV-2 infections in patients, developing a guidance for the management of SAD patients during the COVID-19 pandemic and being able to lead future researches based on the obtained results.

## Patients and Methods

### Online Surveys and Patients

This cross-sectional study was performed by GEAS and was approved by the local Ethics Committee. All outpatients with SAD were eligible. In cooperation with biostatisticians and data-protection specialists to ensure mutual understanding of research objectives and scientifically and legally appropriate data collection, a database-driven online questionnaire was developed and launched on 16 April 2020 known as COVID-GEAS-1. This survey used the google form platform and the target population was patients with SAD. It remained open for 2 weeks and consisted of 28 items on demographic data, systemic autoimmune disease, symptomatology, evolution and healthcare needs, contact with other COVID patients, diagnosis of COVID by nasal swab and other concomitant treatments.

The same online survey (COVID-GEAS-2) was sent on 5 October opened for a period of 15 days.

The database includes nationality, age, detailed rheumatological diagnosis, antirheumatic medication at time of study and changes in the last 3 months. In addition, the contact with COVID patients as well as conducting diagnostic tests and the course and outcome of the SARS-CoV-2 infection are also key parameters. Missing data on diagnosis, outcome and therapies can be queried by directly contacting by mail. Periodic critical evaluation of the registry is carried out by the task force to ensure that the objectives are being met.

In the first survey, only nasopharyngeal swab was included as a diagnosis. In the second cut, the fast antibody test and serological test were included.

### Data Retrieval

Data entered in an electronic case report form with the URL https://es.surveymonkey.com/r/encuestaGEAScovid19 are directly stored by survey-monkey database into an SQL-database on a dedicated server located in Spain and certified according to DIN ISO/IEC 27001 using encryption and secure communication protocols (SSL/TLS and HTTPS). Data entered in these forms are checked for plausibility immediately. Web-forms use dynamic menus and subquestions. Data allowing for identification of individual patients are omitted, and reidentification is only possible via local files. Aragon's ethics committee authorized the survey and it was approved by the Spanish Agency for Medicines and Health Products (AEMPS).

The survey was addressed to national and regional associations of systemic autoimmune diseases such as Systemic Lupus Erythematosus (SLE), Bechet, Scleroderma, Sarcoidosis, APS, and Sjögren's syndrome. Most of them have been informed directly using established dissemination channels of the GEAS. Other systems as twitter and Facebook were used as dissemination channels.

The prevalence of SARS-CoV-2 infection was expressed as the percentage [with 95% confidence interval (CI)] of cases with SARS-CoV-2 infection confirmed by nasopharyngeal swab, fast test and/or serological test on the total number of patients included in the study. The proportion of patients with confirmed SARS-CoV-2 infection in our cohort was compared to those reported for the general population of Spain, using the Fisher exact test. Statistical significance was defined as *P* < 0.05.

### Statistical Analysis

The proportion of patients with confirmed SARS-CoV-2 infection in our cohort was compared to those reported for the general population of Spain, using the Pearson test. Statistical significance was defined as *P* < 0.05. Analysis was performed descriptively using SPSS Statistics v 25.00.

## Results

On 16th of April 2020, the first survey (COVID-GEAS-1) was sent to all SAD association partners, regardless of their treatment, collecting a total of 1,140 responses in a mean time of 8 days (3–13 days). The objective of this survey was to know the association of COVID with SAD patients at the worst epidemic moment of the pandemic in Spain as well as the incidence and severity of the COVID-19. Most patients were female (90.96%); the median age was 45.3 ± 11.4 years. According to the distribution by systemic autoimmune disease, 563 patients (49.3%) had been diagnosed of SLE, 179 (15.7%) SC, 248 (21.8%) SS, 198 APS, 72 SA, 68 BD, 61 vasculitis (VAS), and 35 patients were diagnoses of rheumatoid arthritis (RA).

Regarding the treatment, 666 patients had started hydroxychloroquine treatment at least 3 months prior to the survey and 512 were under active treatment with corticosteroids with a mean dose of 6.18 ± 4.67 mg/day. A total of 458 patients added one or more immunosuppressive agent: 105 patients azathioprine, 165 mycophenolate, 9 cyclophosphamide, 155 methotrexate, 11 leflunomide, 36 tacrolimus and 18 cyclosporine A. Biological treatment had been used in 155 patients in the last 6 months; 52 of them Rituximab, 47 belimumab, 9 tocilizumab, 38 anti-TNF-alpha and other biological treatments in 10 more cases.

At the time of the survey, 80 patients had reported a previous close contact with symptomatic patients and 35 patients with confirm COVID-19 patients. Twenty-four patients presented symptoms compatible with COVID-19 and 101 patients had undergone SARS-CoV-2 test a maximum of 15 days before with 19 positive results (21.6%). A 26.3% (5) of the total positives were asymptomatic. A total of 20 patients required health care, 4 were followed by telephone, 6 required to see their primary care physician, 6 were admitted to the emergency department and 4 required hospital admission, one of them entered to intensive care unit.

The second survey (COVID-GEAS-2) was sent on 5th of October 2020 with a total of 389 responses with the same objective as COVID-GEAS-1 and mean time of responses was 9 days (range 1–26 days). Similarly, many of the patients were women (92.55%) with a mean age of 40 years. A total of 120 patients (30.8%) were diagnosed of SLE, 81 (20.8%) SC, 132 (33.9%) SS, 26 APS, 4 BD, 49 SA, 12 VAS, and 9 RA. Regarding treatment, 135 patients were on immunosuppressive treatment: 25 azathioprine, 47 mycophenolate, 52 methotrexate, 5 leflunomide, 6 cyclosporine A and 1 patient with cyclophosphomide. A total of 49 patients associated biological treatment, 11 rituximab, 13 belimumab, 3 tocilizumab, 16 anti-TNF-alpha and 6 other biological therapies. Of the total of participants, 28 had presented close contact with people with compatible symptoms, 8 of them with COVID-positive patients. A total of 196 tests were performed with 31 positives (8%). Of the positive patients, 21 were asymptomatic, 17 required telephone medical assistance, 10 received assessment by primary care physician, 3 were evaluated in Emergency Department and 1 patient required hospitalization.

Regarding the global characteristics of the two surveys, a total of 1,360 patients (91.1%) were Caucasian and 114 (7.6%) Hispanic. The rest, 19 patients (1.3%), were classified in other ethnicities. According to the distribution by regions, 336 (22.9%) of the patients lived in Madrid, 188 (12.8%) Andalucía, 181 (12.3%) Cataluña, 116 (7.9%) Valencia, 110 (7.5%) Castilla y León, 94 (6.4%) Aragón, 83 (5.7%) Galicia, 77 (5.2%) Navarra, 56 (3.8%) País Vasco, 44 (3%) Asturias, 42 (2.9%) Murcia, 41 (2.8%) Castilla La Mancha, 33 (2.2%) Canarias, 33 (2.2%) Baleares, 30 (2%) Cantabria, 15 (1%) Baleares, 13 (0.9%) Extremadura and 8 (0.5%) La Rioja.

The demographic, clinical, diagnosis and therapies data from both surveys were compared, with the results shown in Table 1.

**Table 1:** Demographic data, clinical characteristics and treatment of the patients of the two surveys.

	**COVID-GEAS-1**	**COVID-GEAS-2**	***p***
Date	April 15-May 15, 2020	October 10-October24, 2020	
Participants number (*n*)	1,140	389	
Days until reply (median-CI)	8 (3–13)	3 (1–8)	<0.0001
COVID (% of total cohort)	19 (1.7%)	31 (8%)	<0.0001
Male (% of the group)	103 (9.1%)	29 (7.5%)	0.340
Median Age in years: mean (SD)	45.4 (11.3)	46.3 (11.0)	0.145
Patients with almost one SARS-CoV-2 test total (%)	230 (20.2%)	184 (47.3%)	<0.0001
Patients with SARS-CoV-2 test +/SARS-CoV-2 total (%)	19 (8.3%)	31 (16.8%)	0.008
Nasopharyngeal swab +/total nasopharyngeal swab	16/218 (7.3 %)	23/158 (14.6 %)	0.023
Fast antibody test +/fast antibody test total	4/20 (20 %)	4/47 (8.5 %)	0.17
Serologic Test +/serologic test total	0/0	14/34 (41.2 %)	–
**Symptoms**, ***n*** **(%)**			
Any symptom	272 (23. 9%)	74 (19%)	0.049
Cough	201 (17.6%)	51 (13.1%)	0.038
Fever > 37.8°C	24 (2.1%)	2 (0.5%)	0.036
Low-grade fever (37–37.8°C)	114 (10%)	18 (4.6%)	0.001
Dyspnea	97 (8.5%)	34 (8.7%)	0.888
Dyspepsia/anosmia	70 (6.1%)	18 (4.6%)	0.269
Diarrhea	166 (14.6%)	43 (11.1%)	0.082
Asthenia	359 (31.5%)	112 (29%)	0.368
COVID-19 asymptomatics patients (%)	5/19 (26.3%)	24/35 (68.6%)	0.004
**Diagnosis** ***n*** **(%)**			
SLE	563 (49.4%)	120 (30.8%)	<0.0001
Scleroderma	179 (15.7%)	81 (20.8%)	0.02
Sjögren syndrome	248 (21.8%)	132 (33.9%)	<0.0001
APS	198 (17.4)	26 (6.7%)	<0.0001
Reumatoid arthritis	35 (3.1%)	9 (2.3%)	0.44
Vasculitis	61 (5.4%)	12 (3.1%)	0.07
Bechet syndrome	68 (6%)	4 (1%)	<0.0001
Sarcoidosis	72 (6.3%)	49 (12.6%)	<0.0001
No SAD	36 (3.2%)	4 (1.0%)	0.026
**Therapy**, ***n***			
Hydroxychloroquine in the past 3 months	666 (58.4%)	180 (46.3%)	<0.0001
Corticosteroids	512 (44.9%)	148 (38.0%)	0.018
Corticosteroids dose in mg/day: mean (SD)	6.2 (4.7)		
Immunosuppressive treatment	458 (40.2%)	135 (34.7%)	0.056
Azathioprine	105 (9.2 %)	25 (6.4 %)	0.089
Mycophenolate	165 (14.5 %)	47 (12.1 %)	0.239
Methotrexate	155 (13.6 %)	52 (13.4 %)	0.909
Tacrolimus	36 (3.2 %)	6 (1.5 %)	0.092
CyA	18 (1.6 %)	6 (1.5 %)	0.960
Leflunomide	11 (1 %)	5 (1.3 %)	0.592
Cyclophosphamide	9 (0.8 %)	1 (0.3 %)	0.223
Biological treatment	153 (13.4 %)	50 (12.9 %)	0.776
Rituximab	52 (4.6 %)	11 (2.8 %)	0.137
Belimumab	47 (4.1 %)	13 (3.3 %)	0.493
Anti-TNF	36 (3.2 %)	19 (4.9 %)	0.114
Tocilizumab	9 (0.8 %)	3 (0.8 %)	0.972
Other biological treatments	10 (0.9 %)	4 (1 %)	0.787
**Contacts and follow-up**			
Close contact with symptomatic patient	84 (7.9%)	20 (7.6%)	0.900
Close contact with COVID-19 patient	45 (4.2%)	14 (5.3%)	0.426
Inpatients number (emergencies and hospitalization)	9/19 (47.4%)	4/31 (12.9%)	0.018
Severe COVID-19 (hospitalization and/or ICU)	3/19 (15.8%)	1/30 (3.3%)	0.285
Need emergency hospital valoration (for suspected COVID-19)	14/54 (25.9%)	5/41 (12.2%)	0.097
Severe disease: need hospitalization and/or ICU (for suspected COVID-19)=	5/54 (9.3%)	1/41 (2.4 %)	0.179
COVID-19 inpatients	3/19	1/31	0.147
No COVID-19 inpatients	2/35	0/10	

*SD, Standard Deviation*.

**Figure 1:**
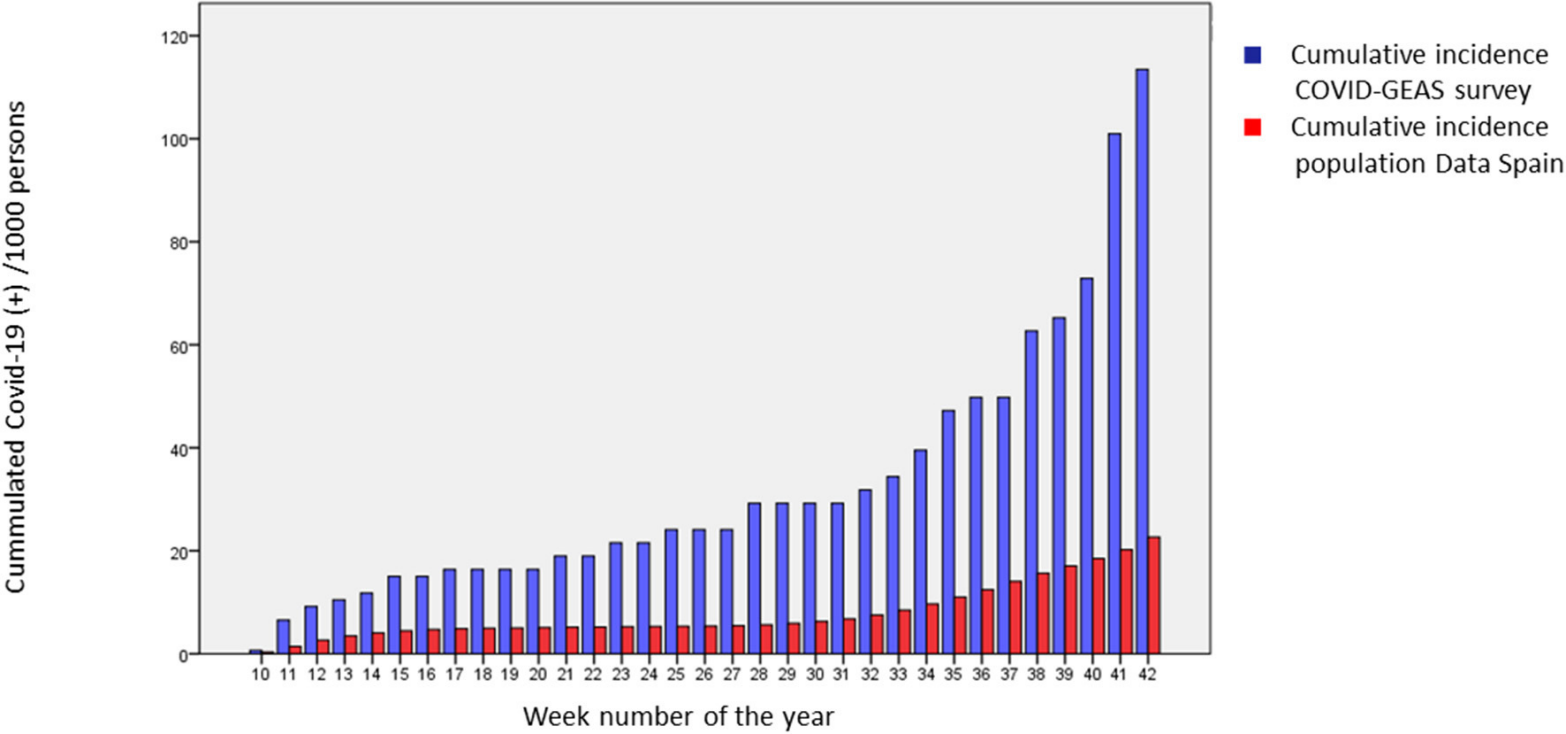
Comparative between cumulative incidence of Spain and COVID-GEAS survey. The proportion of patients with confirmed SARS-CoV-2 infection in our cohort was compared to those reported for the general population of Spain, using the Pearson test.

The joint cumulative incidence of the two surveys was analyzed and compared with the total cumulative incidence in Spain, obtaining results 3.925 times higher in the group of patients with systemic autoimmune diseases (Figure 1).

The patients diagnosed with SARS-CoV-2 infection were jointly analyzed because there were no significant differences in the survival curves (log rank 0.566). By ethnicity, 48 patients were Caucasian and 2 Hispanic. The data were compared with the patients without a confirmed diagnosis of COVID-19 obtaining the results shown in Table 2.

**Table 2:** Comparison of patients diagnosed with SARS-CoV-2 infection.

	**No COVID-19 confirmed**	**COVID-19 confirmed**	***p***
Patients COVID-GEAS-1 (*n*)^*^	1,121 (98.3%)	19 (1.7%)	<0.0001
Patients COVID-GEAS-2 (*n*)^*^	358 (92%)	31 (8%)	<0.0001
Patients COVID-GEAS-1 + GEAS 2: N° (%) (^*^)	1479 (96.7%)	50 (3.3 %)	
Median Age in years (range): mean (SD)	45.2 (11.2)	45.7 (11.9)	0.963
Male (% of the group)	131 (8.9%)	1 (2.0%)	0.058
Male/female	131/1,348	1/49	
Patient with SARS-CoV-2 test total (%)	364 (24.6%)	50 (100%)	<0.0001
Patient with total nasopharyngeal swab	332 (22.4%)	44 (88%)	<0.0001
Nasopharyngeal swab test number: mean (SD)	1.21 ± 0.57	1.89 ± 1.06	<0.0001
Patient with Fast antibody test	58 (3.9%)	9 (18%)	<0.0001
Patient with serological test	18 (1.2%)	16 (32%)	<0.0001
**Symptoms**, ***n*** **(%)**			
Any symptom	322 (21.85)	24 (48%)	<0.0001
Cough	235 (15.9%)	17 (34%)	0.001
Fever > 37.8°C	20 (1.4%)	6 (12%)	<0.0001
Low-grade fever (37–37.8°C)	123 (8.3%)	9 (18%)	0.016
Dyspnea	118 (8%)	13 (26%)	<0.0001
Dyspepsia/anosmia	74 (5%)	14 (28%)	<0.0001
Diarrhea	197 (13.3%)	12 (24%)	0.031
Asthenia	450 (30.4%)	22 (44%)	0.041
**Diagnosis**, ***n*** **(%)**			
SLE	666 (45%)	17 (34%)	0.123
Scleroderma	247 (16.7%)	13 (26%)	0.085
Sjögren syndrome	372 (25.2%)	10 (20%)	0.408
APS	219 (14.8%)	5 (10%)	0.344
Reumatoid arthritis	42 (2.8%)	2 (4%)	0.426
Vasculitis	70 (4.7%)	3 (6%)	0.431
Bechet syndrome	70 (4.7%)	2 (4%)	0.578
Sarcoidosis	117 (7.9%)	4 (8%)	0.569
No SAD	38 (2.6%)	2 (4%)	0.379
**Therapy**, ***n***			
Hydroxychloroquine in the past 3 months	823 (55.6%)	23 (46%)	0.177
Corticosteroids	644 (43.5%)	16 (32%)	0.105
Immunosuppressive treatment	570 (38.5%)	23 (46%)	0.287
Azathioprine	126 (8.5%)	4 (8%)	0.577
Mycophenolate	202 (13.7%)	10 (20%)	0.202
CyA	23 (1.6%)	1 (2%)	0.553
Tacrolimus	41 (2.8%)	1 (2%)	0.597
Cyclophosphamide	10 (0.7%)	0	0.716
Methotrexate	199 (13.5%)	8 (16%)	0.605
Leflunomide	16 (1.1%)	0	0.586
Biological treatment	195 (13.2%)	8 (16%)	
Rituximab	62 (4.2%)	1 (2%)	0.380
Belimumab	60 (4.1%)	0	0.131
Tocilizumab	11 (0.7%)	1 (2%)	0.330
Anti-TNF	50 (3.4%)	5 (10%)	0.013
Other biological treatments	13 (0.9%)	1 (2%)	0.373
**Contacts and follow-up**			
Close contact with symptomatic patient	1,011,290 (7.8%)	3/40 (7.5%)	0.939
Close contact with COVID-19 patient	49/1,290 (3.8%)	10/40 (25%)	<0.0001
Emergency valoration (for suspected COVID-19)	6/45 (13.3%)	13/50(26 %)	0.123
Severe disease: hospitalization and/or ICU (for suspected COVID-19)=	2/45 (4.4%)	4/50 (8%)	0.390

* *Patients with complete data that could be included*.

The 50 patients diagnosed of COVID-19 infection were analyzed. A total of 17 patients (34%) were diagnosed of SLE, 13 (26%) SC, 10 (20%) SS, 5 APS (10%), 2 BD (4%), 2 RA (4%) and 2 did not meet established SAD criteria (4%). Regarding treatment, 23 patients were on hydroxychloroquine treatment (46%) and 16 patients on glucocorticoid treatment (32%). Of all patients infected by COVID-19 23 patients were on immunosuppressive treatment: 4 azathioprine, 10 mycophenolate, 8 methotrexate, 1 cyclosporine A, and 1 patient with tacrolimus. A total of 8 patients associated biological treatment, 1 with rituximab, 1 tocilizumab, 5 anti-TNF-alpha and 1 omalizumab. Regarding evolution, 24 did not need medical attention, 13 were evaluated in Emergency Department and 4 patients required hospitalization, one of them in intensive care unit.

**Figure 2:**
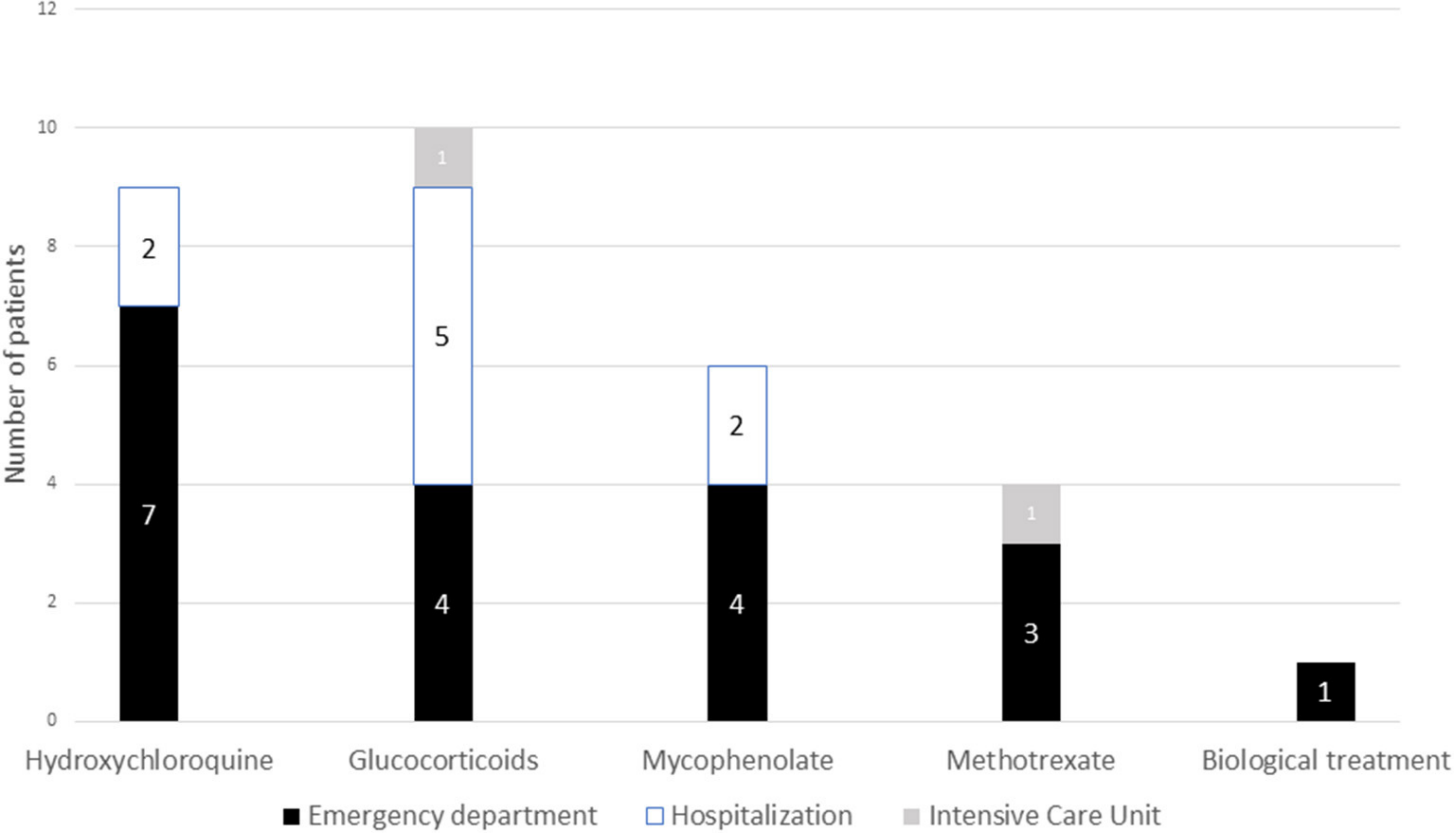
Association between the treatment of systemic autoimmune diseases and severity. Number of patients in each of the immunosuppressive treatments and type of need for hospital care.

Of the 17 patients who required some medical attention, all of them were women. The most frequent SAD was SLE in 6 patients (35.29%) followed by scleroderma in 5 (29.41%), Sjögren's syndrome in 3 (17.65%), 2 Sarcoidosis (11.76%) and one vasculitis (5.88%). A total of 10 (58.8%) patients were in active treatment with glucocorticoids (increasing to 100% of the patients who required hospitalization) and 9 (52.9%) in treatment with Hydroxychloroquine. Eleven patients (64.7%) were on immunosuppressive treatment, the most frequent mycophenolate with 6 patients (35.29%) and methotrexate in 4 patients (23.53%). Only one patient in the emergency department assistance group was under treatment with biological drugs. Detailed results are shown in Figure 2.

Of the total of 666 patients with SLE analyzed, 17 (2.5%) had active infection by SARS-CoV-2. Of the 247 patients diagnosed with SC, 13 (5%) presented confirmation of infection during the follow-up period. In the subgroup of patients with SS, made up of 372 patients, 7 (1.8%) had active infection. Of the 219 patients with a diagnosis of APS, five patients had confirmed infection. Finally, of the rest of the patients analyzed (221) with other SAD, 9 patients presented active infection by SARS-CoV-2.

**Figure 3:**
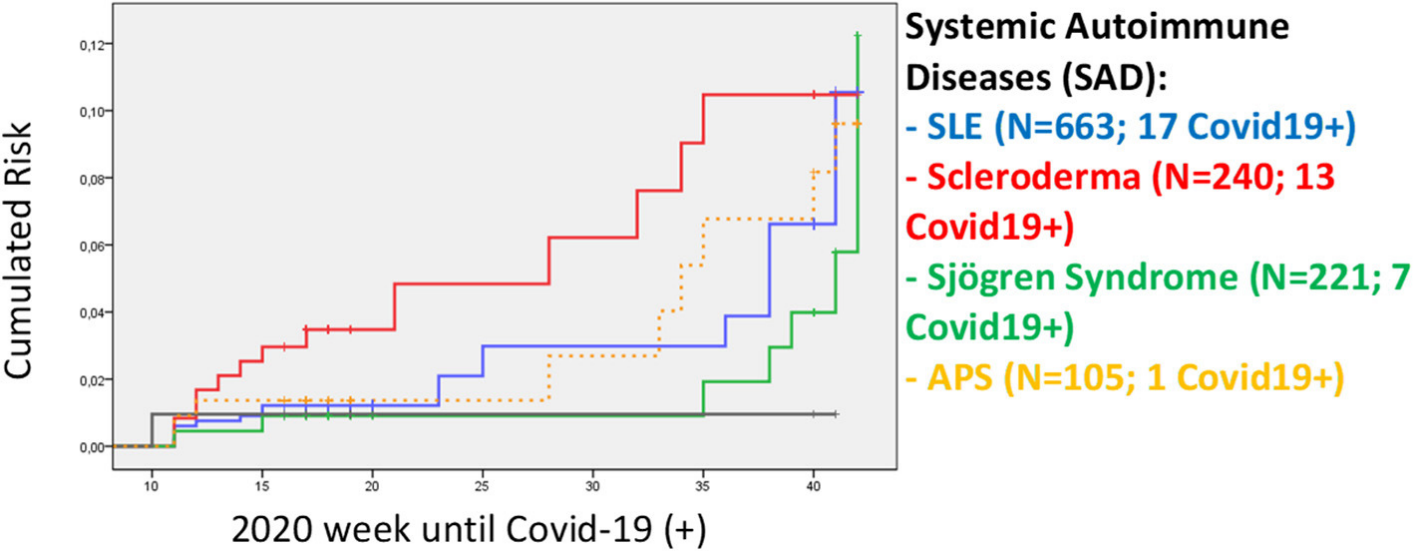
Cumulative incidence of COVID-19 for each of the SAD. Accumulated incidence in the weeks of 2020 for each of the autoimmune diseases. SLE, systemic lupus erythematosus; APS, antiphospholipid syndrome.

The cumulative incidence of COVID-19 for each of the SAD between weeks 10 and 42 of the 2020 was analyzed, obtaining the results obtained in Figure 3.

Multivariate analysis (COX regression) was performed without obtaining differences in any of the SAD. Regarding the treatments, significant differences were found (*p* 0.011) in the treatment with anti-TNF-alpha with OR 3.422 (1.322–8.858). Similarly, a trend to significance (p 0.094) was observed in patients receiving mycophenolate treatment [OR 2.016 (0.996–4-081)]. Of the 60 patients under active treatment with belimumab, none of them had confirmed SARS-CoV-2 infection.

## Discussion

In this study, SARS-CoV-2 infection was evaluated among 1,529 patients with SAD residing in Spain with an incidence of SARS-CoV-2 infection comparable to that observed in other European countries.

Global exact incidence and prevalence in Spain of systemic lupus erythematosus, scleroderma or systemic vasculitis actually is unknown but the incidence is possible <1% of the Spanish population.

The Spanish population reached on January 1, 2020 reached 47.4 million inhabitants. In relation to these data, in Spain there would be a total of almost half a million patients with a diagnosis of systemic autoimmune disease.

Although the sample of patients is small, we do understand that it can help to clarify whether or not this type of vulnerable population is at greater risk of contracting the infection.

Our results are consistent with the data in the literature so far available on COVID-19 and immunosuppressive treatment (4, 7, 11). If it is not protective, at least no warnings suggestive of a pejorative evolution of COVID-19 have been detected. However, these studies do not fully clarify whether or not patients with immunosuppressive therapy are at increased risk of developing severe forms of COVID-19 compared with the general population (12).

In our study, anti-TNF treatment was associated with a >3-fold risk of suffering from SARS-CoV-2 infection, although in all cases it was with mild symptoms. This could suggest that although the risk of infection with this immunosuppressive treatment appears to be higher, the severity of symptoms in all cases was mild, suggesting that the use of TNF-alpha inhibitors could be a potential treatment for acute respiratory failure caused by SARS-CoV-2 infection (13). Similarly, the use of mycophenolate showed a trend to significance with a risk increased twice, which would be similar to what is currently published. Although *in vitro* studies had showed promising results for mycophenolate against SARS-CoV-2 (14), the *in vivo* studies suggest that its use is likely to cause more harm than benefit and hence is not likely to be useful against coronavirus infections (15, 16). Interestingly, no case of SARS-CoV-2 infection was found in the group of patients with belimumab, which could suggest the possibility of the B lymphocyte having some role in SARS-CoV-2 infection. Woodruff et al. (17) found extrafollicular B cell activation in critically ill patients with COVID-19, similar to what has been observed in autoimmunity.

Pablos et al. (18) investigated the prevalence of COVID-19 in seven Spanish hospitals providing medical care for a population of 2.9 million patients and found a comparable prevalence of the infection in SS and SC showed a higher prevalence of SARS-CoV-2 infection in comparison with the general population; in contrast, prevalence in SLE patients was similar to that of the reference population.

Our study shows a cumulative incidence up to four times higher than the general population, although with large discrepancies in each of the autoimmune diseases, mainly due to patients' increased susceptibility to infections, the deeper immune-system impairment and favored by the high exposure to the virus at medical facilities before the restriction measures on individual movement (19). The higher prevalence of COVID-19 in SAD compared to that found in general population was further emphasized by some demographic observations; in particular, ASD patients showed lower mean age, as well as a higher percentage of females. These findings are in counter tendency with respect to the epidemiology of COVID-19 symptomatic patients, which are prevalently male, aged >60 years.

Similarly to the study previously mentioned, patients with SC showed a higher cumulative incidence, as well as patients with undifferentiated connective tissue disease and VAS. In contrast, patients with SS, SLE, and APS did not show a higher incidence. This unexpected discrepancy among SAD patients might be explained at least partly by the age, the higher proportion of females and the different treatment among autoimmunity diseases.

In Pablos et al. study (18), patients with autoimmune disease had an increased risk of intensive care/mechanical ventilation [adjusted OR for mechanical ventilation 3.11 (95% CI: 1.07–9.05), *p* = 0.04]. However, this did not associate a statistically significant higher mortality (6%) or an overall hospital admission rate. However, other comorbidities, disease activity or the use of immunosuppressive drugs were not analyzed. Our study shows that, although the incidence is higher than in the general population, overall the rate of hospital admission, ventilation, or death is much lower than that reported in the general population. These data must be analyzed very cautiously since, as it is an online survey, it is very possible that there could be a selection bias, with less participation of patients who may have presented more severe forms of the disease.

The Global Rheumatology Alliance has established a registry of SAD patients with COVID-19 infection (10). This is an international initiative, supported by ACR and the EULAR with the possibility to include SAD patients affected by COVID-19 from all over the world. This study showed a high rate of hospitalization (46%) and mortality (9%) altogether, with SLE and VAS patients showing a higher propensity to be hospitalized than other patients do. These data are different from those obtained in our study, where the levels of hospitalization and severity are clearly lower than those of the general population. Similarly, treatment with doses >10 mg of prednisone was associated with hospitalization, something that did not happen in our study and that, given that the only treatment established as effective for severe SARS-CoV-2 infection is the use of corticosteroids, it could be very controversial. We did not find a significant association between antimalarial use and hospitalization in adjusted analyses as has been shown in multiple previously published studies the use of hydroxychloroquine was not associated with a decrease in hospitalization (20–22).

Strengths of our study include the first serial large analysis of patients with rheumatic diseases and COVID-19 with patient participation. All case data were entered by patients or their relatives. The registry includes cases from all over Spain suggesting that our findings are more generalizable than single-center or regional studies. Since the registry's inclusion criteria aren't restricted to those with rheumatic disease and COVID-19, that includes the ability to make comparisons with those who do not have COVID-19. Furthermore, the performance of multiple surveys allows a more optimal integration of data from a similar cohort of patients as well as a better representation of the cumulative incidence.

Despite these strengths, there are important limitations to these registry data. The COVID-GEAS registry is voluntary and does not capture all cases of COVID-19 in patients with rheumatic disease.

This approach to data collection places limitations on causal conclusions and temporal relationships and therefore we can only make limited inferences based on our results. It is a survey that has its limitations in the first place, not all patients have access to digital support to be able to carry it out. The digital divide in Spain is high and, above all, it is greater the older the patients.

Patients with more severe disease, admitted to UCI or hospital wards, have possibly not been able to answer this survey. But it can help us to have an idea of the cases of asymptomatic or oligosymptomatic infection and with less serious disease.

There is selection bias due to several factors, including geographic location, hospitalization status and disease severity, with the mild cases most likely to be captured, which quite possibly explains this remarkable increase in cumulative incidence compared to other published series.

Another important limitation is the significant discrepancy from the number of responses between the two surveys. It is possible that there were more responses in the first survey because it coincided with the first wave of infection in Spain and also coincided with the state of alarm and home confinement.

The second survey coincided with the end of the second wave and there was no longer any state of alarm and the experts no longer found themselves trusting. The fact that the patients were trusting in their home makes them have more time to be able to answer these types of surveys.

In terms of diagnostic tests, despite the fact that the nasopharyngeal swab was not as accurate as the fast antibody test and serological test, we didn't include these tests in the first survey, because in Spain these tests were not available in the public health system at the time the survey was carried out.

This series of cases demonstrates that the majority of patients with rheumatic diseases captured in our registry recover from COVID-19 although the real incidence of SARS-CoV-2 infection in autoimmunity patients is much higher possibly, due to the underestimation of mild or moderate cases that do not require specific attention. In some cases, exposure to specific medication classes is associated with lower odds of hospitalization; however, these findings should be interpreted with caution because of a high risk of bias.

## Data Availability Statement

The raw data supporting the conclusions of this article will be made available by the authors, without undue reservation.

## Ethics Statement

Written informed consent was obtained from the individual(s) for the publication of any potentially identifiable images or data included in this article.

## Author Contributions

All authors listed have made a substantial, direct, and intellectual contribution to the work and approved it for publication.

## Funding

This work was supported by SEMAIS.

## Conflict of Interest

The authors declare that the research was conducted in the absence of any commercial or financial relationships that could be construed as a potential conflict of interest.

## Publisher's Note

All claims expressed in this article are solely those of the authors and do not necessarily represent those of their affiliated organizations, or those of the publisher, the editors and the reviewers. Any product that may be evaluated in this article, or claim that may be made by its manufacturer, is not guaranteed or endorsed by the publisher.
